# Néel-like domain walls in ferroelectric Pb(Zr,Ti)O_3_ single crystals

**DOI:** 10.1038/ncomms12385

**Published:** 2016-08-19

**Authors:** Xian-Kui Wei, Chun-Lin Jia, Tomas Sluka, Bi-Xia Wang, Zuo-Guang Ye, Nava Setter

**Affiliations:** 1 Ceramics Laboratory, EPFL-Swiss Federal Institute of Technology, Lausanne 1015, Switzerland; 2 Peter Grünberg Institute and Ernst Ruska-Centre for Microscopy and Spectroscopy with Electrons, Forschungszentrum Jülich GmbH, 52425 Jülich, Germany; 3 The School of Electronic and Information Engineering, Xi'an Jiaotong University, Xi'an 710049, China; 4 Department of Chemistry and 4D LABS, Simon Fraser University, 8888 University Drive, Burnaby, British Columbia, Canada V5A 1S6; 5 Electronic Materials Research Laboratory, Key Laboratory of the Ministry of Education and International Center for Dielectric Research, Xi'an Jiaotong University, Xi'an 710049, China

## Abstract

In contrast to the flexible rotation of magnetization direction in ferromagnets, the spontaneous polarization in ferroelectric materials is highly confined along the symmetry-allowed directions. Accordingly, chirality at ferroelectric domain walls was treated only at the theoretical level and its real appearance is still a mystery. Here we report a Néel-like domain wall imaged by atom-resolved transmission electron microscopy in Ti-rich ferroelectric Pb(Zr_1−*x*_Ti_*x*_)O_3_ crystals, where nanometre-scale monoclinic order coexists with the tetragonal order. The formation of such domain walls is interpreted in the light of polarization discontinuity and clamping effects at phase boundaries between the nesting domains. Phase-field simulation confirms that the coexistence of both phases as encountered near the morphotropic phase boundary promotes the polarization to rotate in a continuous manner. Our results provide a further insight into the complex domain configuration in ferroelectrics, and establish a foundation towards exploring chiral domain walls in ferroelectrics.

## Abstract (web summary)

Flexible rotation of spontaneous polarization at ferroelectric domain walls is predicted in theory but lacks evidence from experiment. Here, Wei *et al.* image a Néel-like domain wall in Ti-rich ferroelectric Pb(Zr_1−*x*_Ti_*x*_)O_3_ crystals, providing insight in exploring chiral domain walls in ferroelectrics.

## Graphical abstract

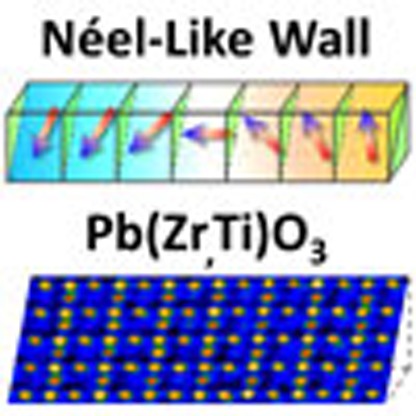

Oxide ferroelectrics, with coupling of polarization, strain, heat and electric field, are widely utilized in electronic devices such as multilayer capacitors, piezoelectric transducers, pyroelectric detectors and non-volatile high-density memories^1^,^2^. While it is well known that properties of the ferroelectrics such as piezoelectric effect and permittivity are substantially enhanced by mobile and stationary domain walls^2^,^3^,^4^,^5^, the ferroelectric domain walls themselves were found recently to possess unique properties that differ thoroughly from the bulk domains, for example, electronic conductivity^6^,^7^,^8^ and photovoltaic effect^9^. These results raise much interest^10^,^11^,^12^ in further exploration of the properties of domain walls and their internal structures.

As known, magnetic 180° domain walls are typically of Bloch type or Néel type^13^,^14^, across which the magnetization vector rotates continuously in a plane parallel or normal to the wall plane, respectively, while ferroelectric 180° domain walls are predominately of Ising type^10^. In spite of this, theoretical and computational studies predict plethora of wall structures in the oxide ferroelectrics, for example, the Bloch-type walls in rhombohedral BaTiO_3_ (refs 15, 16, 17) and tetragonal PbTiO_3_ (ref. 18), where the polarization rotation is enabled by symmetry-lowering at the walls. Driven by the flexoelectric effect^19^,^20^,^21^, a higher-order electromechanical coupling, a Bloch-like component combined with or without a Néel-like component^19^,^22^,^23^ is also predicted at the 180° domain walls. However, the chiral components are negligible in comparison with the Ising component. The recent discovery of ferroelectric monoclinic phases close to the morphotropic phase boundary (PB)^24^,^25^,^26^,^27^, for example, in Pb(Zr_1−*x*_Ti_*x*_)O_3_ (PZT), (1−*x*)PbMg_1/3_Nb_2/3_O_3_−*x*PbTiO_3_ and BiFeO_3_, where the polarization easily rotates within a crystallographic plane, offers new possibilities in the search of new types of domain walls.

Experimentally, direct imaging of light oxygen together with heavy cations using transmission electron microscopy (TEM) serves as the decisive basis to resolve chirality at the ferroelectric domain walls. This requirement is fulfilled by the negative spherical-aberration (*C*_S_) imaging (NCSI) technique in an aberration-corrected TEM^28^. In comparison with the conventional positive *C*_S_ imaging technique, the NCSI technique yields an especially high image contrast^29^. Specifically, the robust image contrast is enhanced by superposition of the amplitude contrast because of electron diffraction channelling with the phase contrast^30^. As a result, atoms appear sharp and bright on a dark background^31^, light and heavy elements can be clearly imaged together and picometre-precision measurements of atomic positions become accessible^32^.

Here, by means of direct imaging of electric dipoles using the NCSI technique combined with the ultrahigh-precision measurements, we report a Néel-like rotation of polarization at monoclinic domain walls in ferroelectric PZT (*x*=0.54, 0.60) single crystals, where the monoclinic order coexists with the tetragonal order at the nanometre scale. The formation mechanism of the domain wall is interpreted in the light of polarization discontinuity and clamping effect at the monoclinic-tetragonal PBs.

## Results

### Phase and domain identification

**Figure 1:**
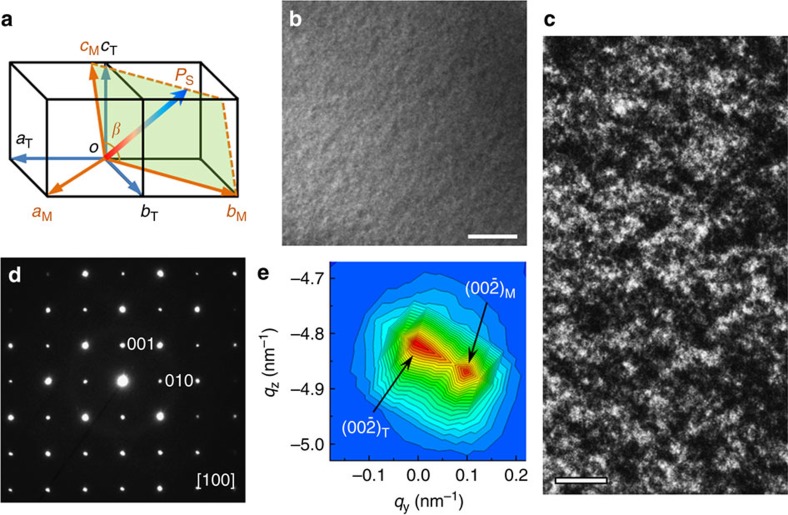
Crystal structures of tetragonal and monoclinic phases and domains in ferroelectric Pb(Zr_0.40_Ti_0.60_)O_3_ crystal. (**a**) Schematic relation of the monoclinic unit cell with respect to the tetragonal unit cells. The spontaneous polarization in the monoclinic phase (space group *C*m) is allowed to rotate flexibly within the (*bc*)_M_ plane as indicated by the arrow *P*_S_. (**b**) Bright-field TEM image illustrating morphology of the crystal specimen. Scale bar, 1 μm (**c**) Dark-field TEM image recorded under two-beam condition using **g**=(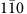)_T_ reflection in the [110] orientated specimen. The bright-contrast regions indicate the monoclinic domains with a polarization component along the [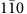]_T_ direction. Scale bar, 20 nm. (**d**) The representative electron diffraction pattern recorded along the [100]_T_ zone axis. The diffraction spots are indexed with respect to the tetragonal structure. (**e**) Reciprocal space mapping of the (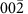) reflection spot from **d** to show the spot splitting for the coexisting monoclinic and tetragonal phases.

Figure 1a shows schematically the basic axes of the monoclinic and the tetragonal unit cell structures. The relations of their lattice parameters are *a*_M_≈*b*_M_≈√2*a*_T_, *c*_M_≈*c*_T_ with a small tilting angle. The spontaneous polarization (*P*_S_) is oriented along the [001]_T_ direction in the tetragonal phase (space group *P4mm*), but in the monoclinic phase, it is allowed to rotate flexibly within the (110)_T_ plane^33^. Our TEM studies reveal that the monoclinic phase coexists with the tetragonal phase in the PZT (*x*=0.54, 0.60) crystals. Figure 1b shows morphology of the *x*=0.60 PZT specimen oriented along the [100]_T_ direction. The observed nanometre-scale ferroelectric domains are in agreement with earlier experimental results on the second-order nature of the ferroelectric phase transition in this composition range^34^ (Supplementary Note 1 and Supplementary Fig. 1). Under two-beam conditions, diffraction contrast analysis using **P·g** >0 relation (**P** is a component of **P**_**S**_ and **g** is a scatting vector)^35^ reveals that sizes of the nesting domains are less than 20 nm and the domain boundaries cannot be clearly identified, as seen in Fig. 1c. In the selected area electron diffraction pattern shown in Fig. 1d, the reflection spots can be registered as the tetragonal phase, with a tetragonality *c*/*a*≈1.043. However, splitting of the reflection spots, which is thoroughly different from that of 90° domains^36^, can be found by a careful inspection, further corroborating the coexistence of the monoclinic phase with the tetragonal phase.

To clearly reveal the spot splitting, the area including the (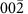) reflection is magnified and exemplified in Fig. 1e. Measurements yield that the *c* axis relating to the left- and right-side reflection spots is ∼0.415 and 0.411 nm, respectively. The good agreement of the measured results with the data from high-resolution synchrotron X-ray diffraction^25^ indicates that the two split spots can be indexed as the tetragonal (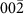)_T_ and the monoclinic (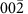)_M_, respectively. The (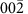)_T_ spot is found to stretch towards the (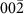)_M_ spot, suggesting that lattice distortion takes place.

### Atom-resolution TEM study

**Figure 2:**
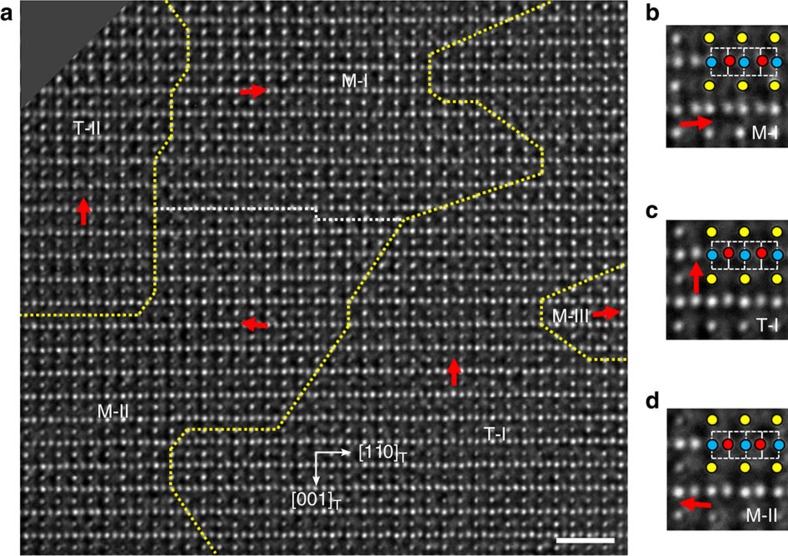
Atom-resolved phase boundaries and domain walls in Pb(Zr_0.40_Ti_0.60_)O_3_ crystal. (**a**) The panoramic view of nesting tetragonal and monoclinic domains viewed along the [110]_T_ direction. Yellow dotted lines trace the boundaries between the tetragonal and monoclinic phases, and white dotted lines trace the domain walls in the monoclinic phase. The phase and domain boundaries are differentiated by mapping the relative displacements of O2 columns (red arrows) with respect to centres of the nearest neighbouring Zr/Ti columns over the whole image. Scale bar, 1 nm. (**b–d**) Enlarged view of the relative displacements of the O2 columns in domains M-I, T-I and M-II in **a**. The colour circles overlapped on the images denote different column types: Pb/O1—yellow, Zr/Ti—blue and O2—red. The vertical dashed and solid lines indicate positions of the Zr/Ti columns and centres of their nearest neighbours. The relative displacements of O2 columns with respect to these centres can be directly identified in this way.

To investigate details of the domain and domain wall structures, high-resolution TEM experiments were performed on the PZT crystals under the NCSI conditions. Figure 2a shows an atom-resolution image recorded along the [110]_T_/[100]_M_ direction of the *x*=0.60 crystal. Considering the relative shifts of oxygen columns with respect to centres of the nearest neighbouring Zr/Ti columns, this image area can be divided into several different domains. Further consideration of the symmetry-allowed directions for the atomic displacements allows us to assign the domain types, either tetragonal or monoclinic, the PBs and the domain walls.

The tetragonal domains, T-I and T-II, show upward shifts of the oxygen columns (denoted by red arrows) along the [001] direction. In contrast, in the monoclinic domains, M-I and M-II, the oxygen columns shift to right-up and left-up, respectively, evidently deviating from the [001] direction. A small part of M-III domain is also detected in the right part of the image area. The displacement features of oxygen columns in the M-I, T-I and M-II domains can be directly identified from the magnified images shown in Fig. 2b–d. Yellow, blue and red circles denote Pb/O1, Zr/Ti and O2 atom columns, respectively. It is seen that the main part of the PBs lies in the (001)_T_, (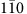)_T_ and (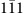)_T_ planes, and the rest is faceted irregularly. Particularly, the M-II, T-II and M-I domains form a flux-closure structure.

**Figure 3:**
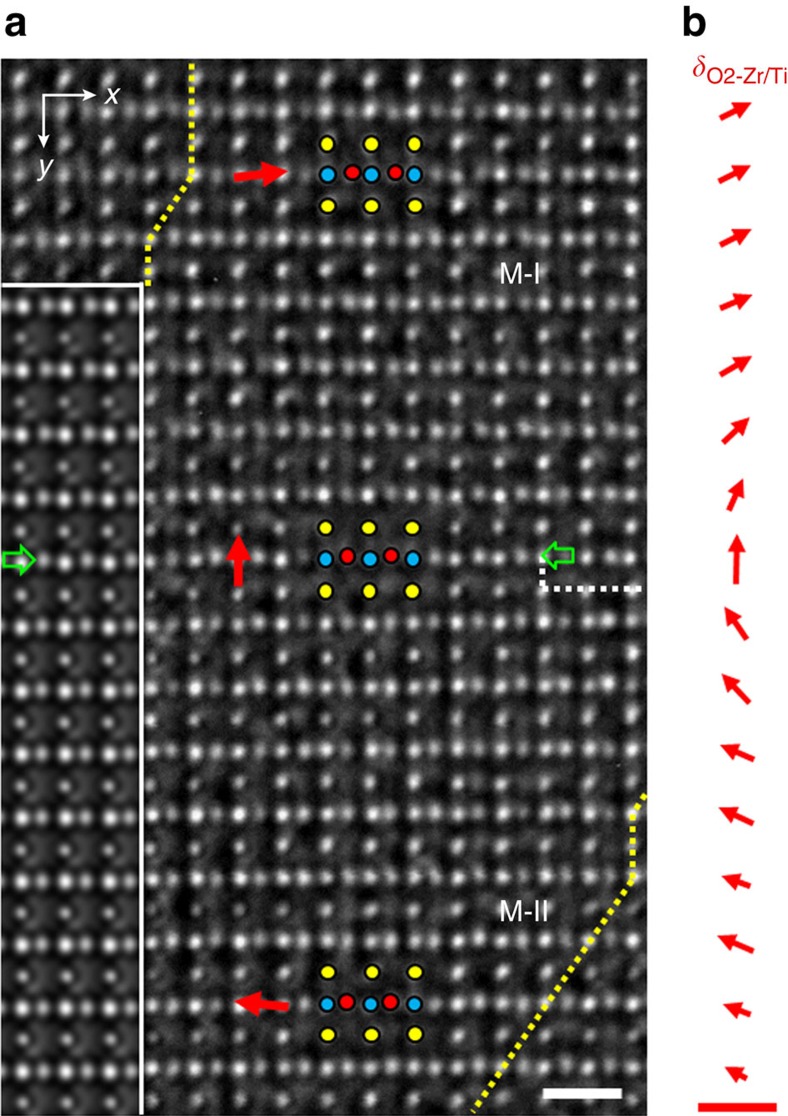
Néel-like continuous rotation of oxygen displacements across the monoclinic domain wall. (**a**) Experimental image of the monoclinic domain wall viewed along the [100]_M_ direction. Green arrows denote the central plane of the domain wall. The inset on the left hand shows a simulated image for thickness of 4.4 nm and defocus of 5.5 nm. Scale bar, 0.5 nm. (**b**) Continuous rotation of the O2-displacement vectors across the domain wall compiled by *δx*_O2-Zr/Ti_ and *δy*_O2-Zr/Ti_ extracted from the structure model used for image simulation. Scale bar, 30 pm.

A particularly interesting feature of the atomic structure is seen in the region including the central plane of the wall between domains M-I and M-II, as denoted by a white dotted line in Fig. 2a and shown again by green arrows in Fig. 3a, where the oxygen columns exhibit vertical displacements. Meanwhile, a wall segment shifted downward by *c*_M_/2, in which the displacements of oxygen columns undergo a sharp transition, is observed at the right side of the region. Measurements of the relative displacements of oxygen columns on the left-side wall segment, extending the areas upwards and downwards from the central plane, reveal a continuous change of the oxygen displacements, that is, continuous rotation of the displacements from leftward (slightly up) in M-II through upward at central plane to rightward (slightly up) in M-I. The displacement feature of oxygen atoms forms a structure similar to the Néel wall in ferromagnets^37^. Quantitative measurement of the atom displacements is performed based on an iterative procedure for comparison between the experimental image and image simulated over this area (Supplementary Note 2 and Supplementary Fig. 2). The simulated image with the best fitting to the experimental image is attached to the left side of Fig. 3a.

**Figure 4:**
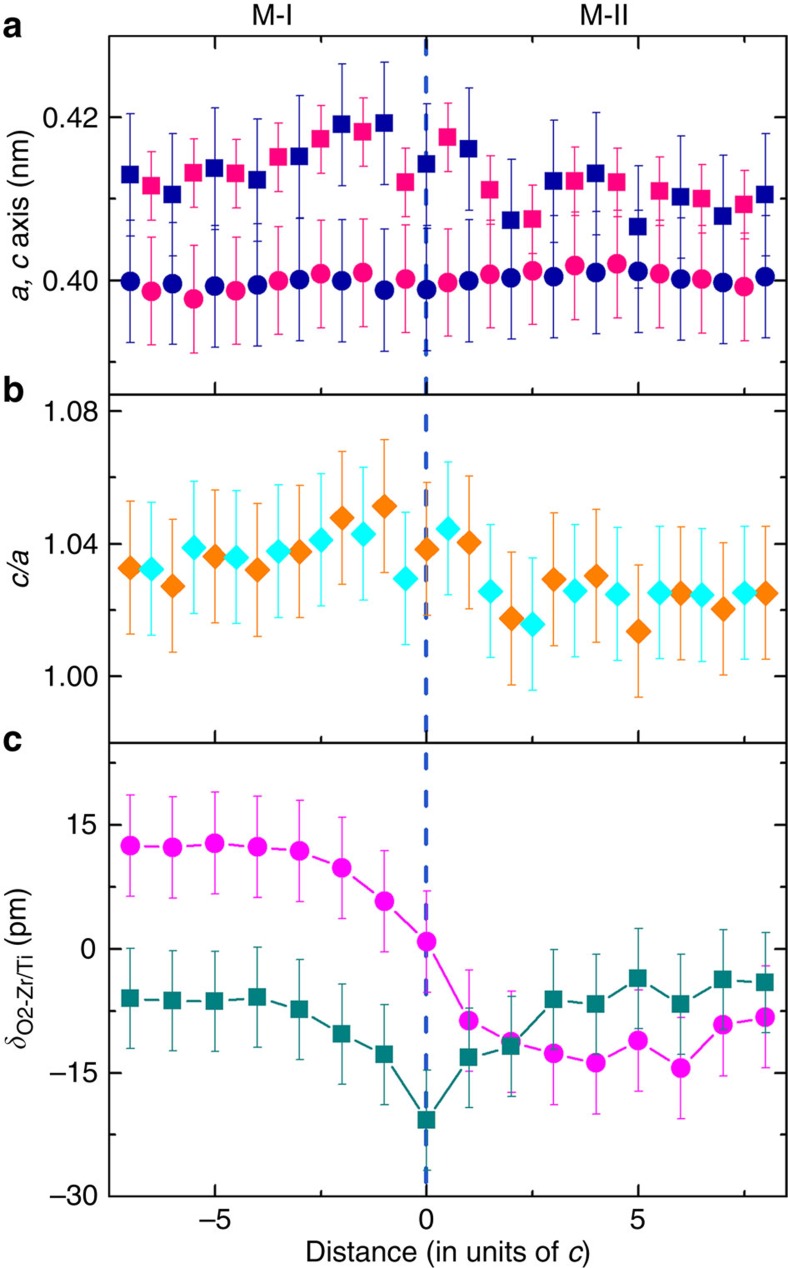
Structural parameter and atomic displacement changes across the Néel-like domain wall. Circles and squares denote the parameters parallel and normal to the wall plane, respectively. (**a**) The *a* axis and *c* axis lattice parameters. Blue and pink symbols represent the values measured from the Pb to Pb and from the Zr/Ti to Zr/Ti atom positions, respectively. (**b**) Tetragonality *c/a* calculated from the *a* axis and *c* axis. (**c**) Relative displacements of the O2 atoms parallel and normal to the domain wall plane. The error bar is the mean s.d. measured from the experimental image and averaged along the wall plane direction.

Figure 4a shows the lattice parameter changes as a function of distance away from the central plane of the Néel-like wall, which are measured from the positions of Pb to Pb atoms (blue symbols) and Zr/Ti to Zr/Ti atoms (pink symbols), respectively. It is seen that the *c* axis in the M-I and M-II domains is 0.415 and 0.410 nm, respectively, which decreases in a stepwise manner across the wall centre. On the contrary, the *a* axis in the M-I domain is ∼0.40 nm, slightly smaller than that in the M-II domain. As a result, the *c*/*a* ratio in the M-I domain is 1.038 in average, it reaches a value of 1.048 near the central plane of the wall and extends into the M-II domain by 2 unit cells. This ratio decreases to ∼1.024 in the rest part of the M-II domain as shown in Fig. 4b. Our statistical studies on both PZT crystals indicate that the *c/a* ratios in the M-I and M-II domains are not necessarily different from each other. It is therefore reasonable to speculate that, to a certain degree, their tetragonality values rely on conditions of the surrounding domains.

With respect to the centres of the nearest neighbouring Ti/Zr atom columns, the rightward horizontal displacements of the O2 atoms (*δx*_O2-Zr/Ti_) undergo a smooth reduction from ∼12 pm in the M-I domain to zero at the central plane of the wall (Fig. 4c). The displacements switch to the opposite direction (leftwards) inside the M-II domain and further increase to 14 pm. Along the vertical direction, the displacements of O2 atoms gradually decay from the maximum of *δy*_O2-Zr/Ti_≈−21 pm at the central plane of the wall towards far ends of the two domains. Since the unit cell dipole moment is proportional to the relative displacement, the data in Fig. 4c represent the transition behaviour of the polarization across the domain wall. Along the horizontal wall plane direction in Fig. 3a, the parallel component of polarization smoothly decreases to zero at the central plane of the wall, and then increases when moving away from the central plane in domain M-II. Along the normal direction of the wall plane, the normal component of polarization reaches the maximum value and then gradually decays towards far ends of the two domains. The change of the relative displacements is shown as vectors in Fig. 3b, demonstrating the clear feature of the Néel-like wall. From the variation of the relative displacements, we infer that width of the domain wall is nine unit cells, four unit cells in each monoclinic domain and one unit cell at the wall centre.

## Discussion

So far, structural investigations of the PZT solid solutions at ambient pressure have revealed the monoclinic (*C*m) phase only, in coexistence with either the rhombohedral phases^33^,^38^,^39^ or the tetragonal phase^40^. Indeed, the coexistence of the monoclinic and the tetragonal phases is evidenced in the present study. To minimize the depolarization field energy^41^,^42^, the polarization vectors in the M-I and M-II domains are confined to lie in the same crystal plane. Therefore, the polarization observed in Fig. 3a is fully projected on the (110)_T_ plane. The formation of the Néel-like wall configuration can be attributed to effects of polarization discontinuity at the PBs and local clamping, that is, to the local electrostatic and elastic conditions in the PZT crystals with coexistence of the two intertwining phases at the nanometre scale.

Our estimation yields a spontaneous polarization value of *P*_S_≈47 μC cm^−2^ for the monoclinic phase and *P*_S_=78 μC cm^−2^ for the tetragonal phase. Neutron diffraction refinement on *x*=0.60 PZT ceramics^40^ reported that the proportion of the two phases is ∼9:11. On the basis of this, the calculated overall polarization for the PZT (*x*=0.60) crystal (*P*_S_=64 μC cm^−2^) is in a good agreement with the macroscopic electric hysteresis loop measurement (Supplementary Note 3 and Supplementary Fig. 3). Owing to the differences in modulus and directions of the polarization with respect to the PB orientation, the polarization discontinuity inevitably results in either positive or negative charging of the PBs.

**Figure 5:**
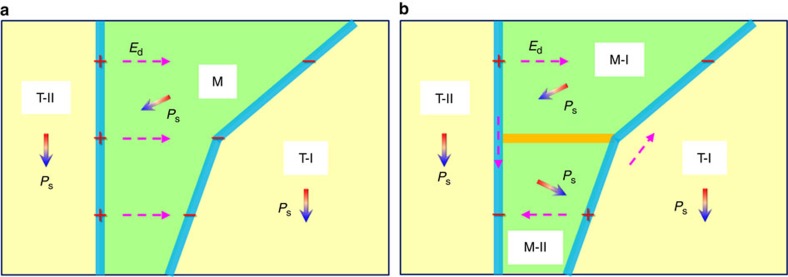
The effect of electrostatically incompatible phase boundaries on formation of the monoclinic domain wall. (**a**) A monoclinic stripe domain is clamped by two tetragonal domains from both sides, where a depolarization field (*E*_d_) is created between the electrostatically incompatible and therefore partly charged PBs (solid blue lines). (**b**) The clamped monoclinic domain is divided into two monoclinic domains, M-I and M-II, to reduce the electrostatic energy associated with the depolarization field in this local area. This results in presence of the domain wall (solid yellow line) in the monoclinic phase. The symbols (+ and −) denote the positive and negative bound polarization charges, respectively.

The cartoon of a single monoclinic domain positioned between the two tetragonal domains in Fig. 5a illustrates the PB charging as a source of the depolarization field *E*_d_. The depolarization field points against the spontaneous polarization and promotes the domain splitting in order to reduce the electrostatic energy (Fig. 5b). It is qualitatively similar to the behaviour of a ferroelectric layer between two dielectric bodies, where alternate charge types on a charged plane produces in average smaller depolarization field, and hence smaller electrostatic energy than a constantly charged plane. In addition, the stray depolarization field produced by the left PBs has a component parallel to the spontaneous polarization at the wall centre. The right PBs promote the same effect with an opposite sign, but have smaller magnitude because of the multiple facets of the boundaries (Fig. 2a). Thereupon, the left-side depolarization field may contribute to drive the polarization to rotate via the [001]_T_ direction. This is manifested by the increased *c/a* ratio near the wall centre, which favours the polarization to lie along the [001]_T_ direction^43^. Similar effects have been reported in ferroelectric thin films^44^,^45^, where flux-closure domains and vortex nanodomains are observed near the interfaces.

**Figure 6:**
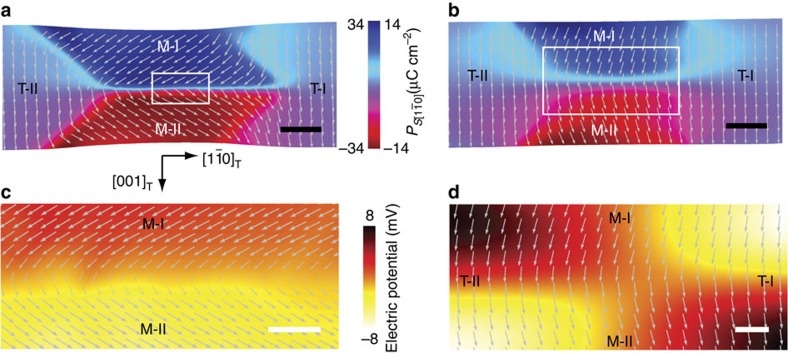
Phase-field simulation of the clamping effect on controlling the monoclinic domain wall width in the multiphase structure. (**a**) The narrow monoclinic domain wall with a sharp polarization transition under an average compressive strain of −0.2%. Scale bar, 5 nm. (**b**) The wide monoclinic domain wall with a continuous rotational polarization transition under an average compressive strain of −0.6%. Scale bar, 5 nm. The colour scales the *P*_S_ component distribution along the horizontal [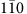]_T_ direction. (**c**) The electric potential distribution in the zoomed region in **a** associated with the sharp polarization reorientation near the wall centre. Scale bar, 0.5 nm. (**d**) The electric potential distribution in the zoomed region in **b** associated with the continuous polarization rotation near the wall centre. Scale bar, 2 nm. The white arrows represent the spontaneous polarization **P**_**S**_.

A three-dimensional (3D) phase-field simulation on a thin (110)_T_ PZT lamella with the energy potential^46^ that allows the presence of a stable monoclinic phase supports the above scenario (see Methods). In addition to the effect of the electrostatic energy, the local elastic conditions are found to play a role in controlling the width of the monoclinic domain wall. When the lamella is compressed with an average strain of −0.2% for all domains in the [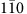]_T_ direction as shown in Fig. 6a, or free of external force, the monoclinic wall is very narrow (approximately one unit cell) and the polarization undergoes a sharp transition at the wall centre. However, as the compressive strain increases to −0.6% (Fig. 6b), the monoclinic wall is substantially broadened to >20 unit cells and the polarization continuously rotates across the domain wall. Together with the electric potential distribution, the polarization rotation behaviours near the monoclinic walls are illustrated in Fig. 6c,d, respectively.

It is well established that sufficiently high content of the larger Zr cations in the tetragonal PZT crystal near the morphotropic PB flattens the potential profile of the system. As a result, the monoclinic states become local minima and the presence of stable monoclinic phases are expected. Although the lower-energy phase in an idealized phase-field model would always sweep-out the higher-energy phase, even if the energy difference is very small, the realistically inhomogeneous Zr distribution, as corroborated by our phase-field simulation, enables the multiphase coexistence in the PZT crystals (Supplementary Note 4, Supplementary Fig. 4 and Supplementary Fig. 5). Facilitated by the shallow Mexican-hat-type energy potential, the energy cost of a domain wall becomes low. Polarization rotation between different stable sites^46^, as shown by the Néel-like rotational changes of the oxygen atoms in Fig. 3b, can therefore occupy large distance when passing through the monoclinic domain wall.

In recent years, the 180° stripe domains^42^,^47^, flux-closure domains^48^,^49^ and vortex nanodomains^45^ have been reported in ferroelectric PZT, BaTiO_3_ and BiFeO_3_. In contrast to these complex domain patterns, for example, the flux-closure structure consisting of consecutive 180°–90°–180° domains, the polarization transitions at these non-180° walls are sharp and the 180° walls are all Ising-like. However, the Néel-like domain walls observed in this work possesses the genuine chiral feature.

In conclusion, our results provide direct experimental evidence for the existence of Néel-like domain walls in ferroelectric materials. The findings highlight that in the low-symmetry ferroelectric phases, which widely exist in solid solutions^27^,^33^ and thin films^26^,^50^, new types of domain walls are yet to be explored both theoretically and experimentally. Extrapolating from the potential utilization of controlled chirality at magnetic Néel walls^14^,^51^ and the control of the polarization configuration by magnetic field^52^, we hope that our findings will stimulate explorations of ferroelectric chiral domain walls and their possible application in future electronic devices.

## Methods

### Material preparation

Lead zirconate titanate crystals were grown by a top-seeded solution growth technique. Optical microscopic examination showed a good optical quality of the crystals without inclusions of visible defects. The good quality of the crystals is supported also by the square-shaped saturated ferroelectric hysteresis loop and the high remnant polarization (Supplementary Fig. 3). The lamella specimens used for TEM investigations were prepared using focused ion beam system. The NanoMill Model 1040 System operated at 500 voltages was used to clean contamination on sample surfaces and mill down the lamella samples.

### Imaging experiments

The TEM investigations were performed on an FEI Titan 80–300 microscope with a *C*_S_ corrector for the objective lens^53^. The available point resolution is better than 80 pm at an operating voltage of 300 kV. Other TEM characterizations were carried out on an FEI Tecnai F20 Microscope. The experimental image was filtered to minimize the effect of contrast noise^32^. Structure modelling and image simulation were carried out using the CrystalKit-MacTempas Software package to determine the relative displacements between different atomic columns. The effective charges used for calculating the spontaneous polarization was inferred from the first-principles calculations^43^.

### Phase-field simulation

The phase-field simulation results (Fig. 6 and Supplementary Fig. 4) were obtained by numerical solution of coupled 3D versions of the time-dependent Landau–Ginzburg–Devonshire equation, Poisson's equation and equation of motion in elastic materials^54^ with eigthth order Landau energy polynomial^46^, which allows presence of the monoclinic phase. Gradient energy coefficients were used 10^2^ times smaller those than in ref. 54 to obtain realistic domain wall widths. Ideal dielectric and compositional inhomogeneity were assumed. Equations were solved on a 2 × 15 × 60 nm^3^ (110)_T_ lamella of PZT crystal. The central 40% of the lamella has *x*=0.5, the rest *x*=0.6 (Supplementary Fig. 4). The (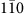)_T_ external boundaries are mechanically clamped and used to stretch and compress the lamella with sinusoidal boundary displacement in the [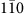]_T_ direction. All other boundaries are mechanically free. The (001)_T_ surfaces have constant potential and all remaining surfaces have zero free charge. Initial conditions were set in the proximity of the expected stationary solution. The model is numerically solved by the finite element method with a time-dependent solver in COMSOL 4.3a (COMSOL Inc., 2012) until stationary solution at each strain was reached.

### Data availability

The data that support the findings of this study are available from the corresponding author upon request.

## Additional information

**How to cite this article**: Wei, X-K. *et al.* Néel-like domain walls in ferroelectric Pb(Zr,Ti)O_3_ single crystals. *Nat. Commun.* 7:12385 doi: 10.1038/ncomms12385 (2016).

## Supplementary Material

Supplementary Information Supplementary Figures 1-5, Supplementary Notes 1-4 and Supplementary References
