# Supplementary material for: Néel-like domain walls in ferroelectric Pb(Zr,Ti)O3 single crystals
Source: Nat Commun. 2016 Aug 19;7:12385. doi: 10.1038/ncomms12385 (PMC4992163; doi:10.1038/ncomms12385)
Supplement: Supplementary Information — Supplementary Figures 1-5, Supplementary Notes 1-4 and Supplementary References [file ncomms12385-s1.pdf]

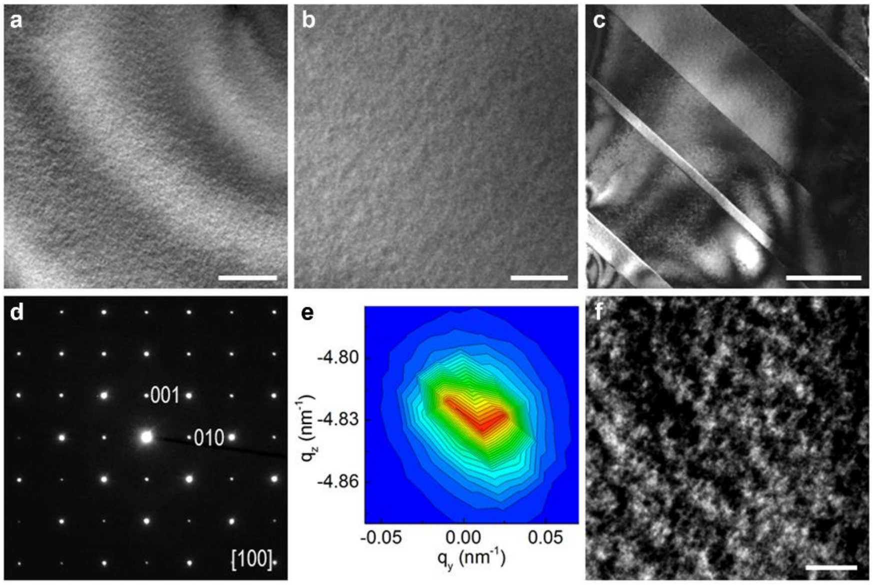

Supplementary Figure 1 Domain morphology of different $Pb(Zr_{1-x}Ti_x)O_3$ crystals. a-c. Bright-field image of the $Pb(Zr_{1-x}Ti_x)O_3$ (PZT) crystals with composition x = 0.54, 0.60 and x = 0.65, respectively. Scale bars, 0.5 μm, 1μm and 1μm. **d-f.** The electron diffraction pattern, reciprocal space mapping of the $(00\bar{2})$ reflection and dark-field image recorded under two-beam condition using **g** = $(1\bar{1}0)_T$ reflection in the [110] orientated specimen in the x = 0.54 PZT crystal. Scale bar, 50 nm.

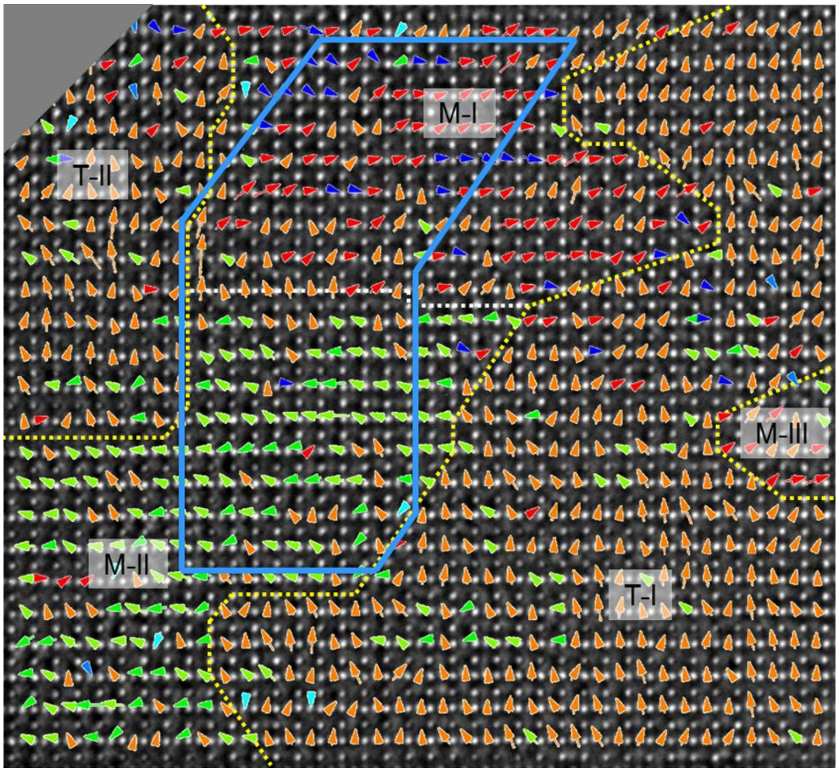

Supplementary Figure 2 The mapping data of $\delta_{O2\text{-}Zr/Ti}$ overlapped on the NCSI image. The length of the arrow indicates their displacement magnitude, and their color indicates their displacement directions. The yellow dotted lines denote the phase boundaries, the white dotted lines denote the monoclinic domain walls. The blue solid lines highlight the image area used for quantitative image simulation and analysis, corresponding to Fig. 3b and Fig. 4.

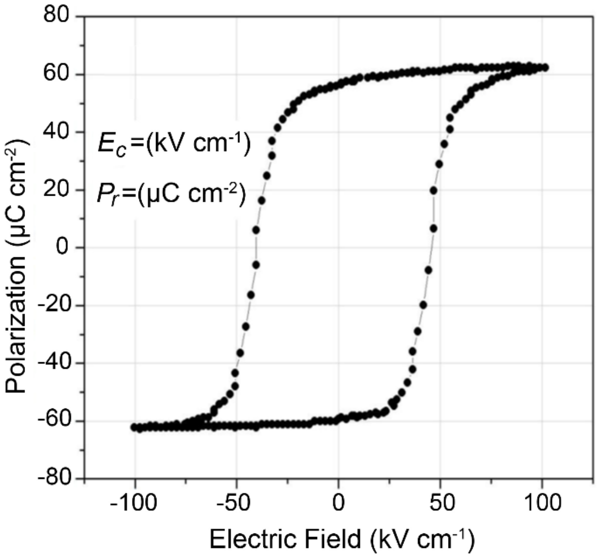

Supplementary Figure 3 Polarization-electric field hysteresis loop of the x = 0.60 PZT crystal. The measurement was performed at room temperature on a pseudo-cubic (001) platelet by means of a standardized ferroelectric testing system (RT66A, Radiant Technologies).

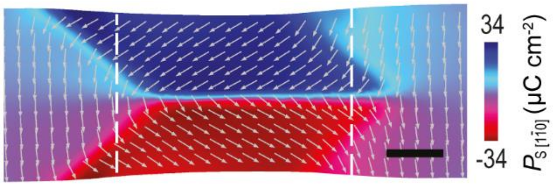

Supplementary Figure 4 Phase-field simulation of coexisting monoclinic and tetragonal phases realized by introducing inhomogeneous Zr distribution. The dashed lines indicate the composition boundaries between the monoclinic phase with x = 0.5 in the central region and the tetragonal phase with x = 0.6 in the bilateral regions. The color scales the P_S component distribution along the horizontal $[1\bar{1}0]_T$ direction.

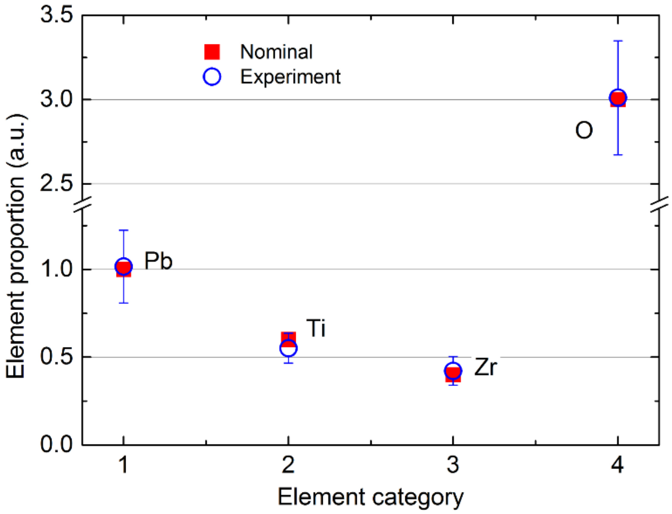

Supplementary Figure 5 The element proportion detected by energy dispersive X-ray spectroscopy on the x = 0.60 PZT crystals. The sizes of the detection spots are dozens of nanometers. The measurement error is s.d. of the element proportion averaged from dozens of detection spots.

Supplementary Note 1 Phase and domain structure in the $Pb(Zr_{1-x}Ti_x)O_3$ crystals

In our experiments, $Pb(Zr_{1-x}Ti_x)O_3$ (PZT) crystals with compositions of x = 0.54, 0.60 and 0.65 are investigated using transmission electron microscope (TEM). Morphology of the specimens shown in Supplementary Figs. 1a and 1b reveals that for x = 0.54 and 0.60 PZT crystals, the macroscopic symmetry breaking leads to coexistence of monoclinic and tetragonal domains at nanometer scale. This indicates that the ferroelectric-to-paraelectric phase transition is of the second order for these two samples[1,2]. In contrast, the mesoscopic stripe domains, with width of hundreds of nanometers and length up to micrometers, observed in the x = 0.65 PZT crystal suggests a first-order phase transition, see Supplementary Fig. 1c. This agrees with the previous experimental finding that the tricritical point, i.e., a meeting point of the first-order with the second-order phase transition in the composition-temperature phase diagram, located at x = 0.62 in the PZT solid solutions[3].

Supplementary Fig. 1d shows the electron diffraction (ED) pattern recorded along the [100] direction. Similar to the x = 0.60 PZT crystal, splitting of the reflection spots reveals coexistence of the monoclinic phase with the tetragonal phase in the x = 0.54 PZT crystal. Specifically, the spot splitting of the $(00\bar{2})$ reflection is illustrated via mapping the intensity distribution in Supplementary Fig. 1e. Measurements on the ED pattern reveal that the *c*-axis for the tetragonal and the monoclinic phase is $c \approx 0.415$ nm and 0.414 nm, respectively. Under two-beam conditions, the domain distribution in the x = 0.54 crystal is investigated. As Supplementary Fig. 1f shows, the monoclinic domains coexist with the tetragonal domain at nanometer scale. This behavior is similar to that in the x= 0.60 PZT crystal.

Supplementary Note 2 Image quantification

Concerning the quantitative measurements of the experimental image, firstly, the position of each atomic column is determined by a least-squares fit of two-dimensional gauss profiles to each of the individual intensity maxima in the image[4]. Based on this, the *c*- and *a*-axis lattice parameters as well as the displacement parameters $\delta x_{\mathrm{O2\text{-}Zr/Ti}}$ and $\delta y_{\mathrm{O2\text{-}Zr/Ti}}$ away from centers of the nearest Zr/Ti columns are determined for each of the unit cell. Supplementary Fig. 2 shows the mapping result of the relative displacements of oxygen columns over the whole image area of Fig. 2a. The blue solid lines highlight the image area covering the monoclinic domains and the Néel-like domain wall, over which the quantitative image simulation and analysis is carried out. This is the region with the data corresponding to Fig. 3b and Fig. 4. Since we are only interested in the parameter changes as a function of the distance away from the domain-wall center, mean values of the parameters are obtained by averaging the displacements along the wall plane direction in highlighted area. The mean measurement error for all column types is less than 6 pm.

Secondly, for ultrahigh precision measurements, deviation of the contrast maxima in the experimental image from their real atomic positions, which originates from the residual objective-lens aberrations and the unavoidable tilting of the specimen from the incident electron beam, is eliminated by means of iteratively comparing the calculated image with the experimental image. The iterative comparison was carried out through adjusting the imaging parameters on the basis of a tetragonal domain region, part of *T*-I domain in Fig. 2a, until the best fit between the calculated and the experimental image was reached. Here, the relative displacements of Zr/Ti and O2 columns with respect to centers of the Pb sublattices are considered to determine the imaging conditions. The final imaging parameters were determined as the experimental imaging conditions: specimen tilting 4.2 mrad (-30 degree), $A1$ = 17 Å, $A2$ = 800 Å, $B2$ = 500 Å. The lattice parameters for the

tetragonal phase are $a = b = 4.0000$ Å, $c = 4.1567$ Å, and the atomic positions are Pb (0.00, 0.00, 0.00), Zr/Ti (0.50, 0.50, 0.528), O1 (0.50, 0.50, 0.12), O2 (0.50, 0.00, 0.578). The calculated spontaneous polarization is $P_S = 78$ μC cm^{-2}, which is in good agreement with the first-principles calculation $P_S = 78$ μC cm^{-2} (Ref. 5).

Thirdly, a structural model for the Néel-like wall region was constructed for the image simulation based on the determined imaging conditions. An additional procedure of iterative comparison of the parameters, i.e., the *c*-, *a*-axis lattice parameter and the displacement parameter $\delta x_{\mathrm{Zr/Ti}}$, δx_{O}, $\delta y_{\mathrm{Zr/Ti}}$ and δy_{O}, from the calculated image with the mean values of the parameters from the experimental image was carried out through adjusting the atomic positions until the experimental parameters were best fitted by the calculated ones. The structure model leading to the calculated image with the best fit to the experimental image provides the real atomic positions of the Néel-like wall region.

Since the O1 atoms are not directly visible along the viewing direction, which overlap with the Pb atoms, their displacements parallel to the wall plane is assumed as $\delta x_{\mathrm{O1}} \approx \delta x_{\mathrm{O2\text{-}Zr/Ti}}$ in our image simulation. Since the displacements of O2 atoms in the unit cells of the wall center are the same as the referred tetragonal unit cells, whose O1 atom positions are adopted by the unit cells at the wall center. Following this initialization, the relative displacements of O1 atoms with respect to the Pb atoms are assumed to decay from the wall center to the monoclinic domains, in a manner mimicing the variation trend of O2 atoms ($\delta y_{\mathrm{O2\text{-}Zr/Ti}}$) along the normal direction of the wall plane. According to these atomic displacements and Born effective charges[6], our calculation estimates that the polarization for the monoclinic phase is $P_S \approx 47$ μC cm^{-2}.

From the mapping data of $\delta_{\mathrm{O2\text{-}Zr/Ti}}$, i.e., the arrows overlapped on Supplementary Fig. 2, it can be seen that the transition region of relative displacements across the phase boundaries is

normally very narrow, at sub-unit cell scale. This suggests that the flexoelectric effect near the phase boundaries is negligible and their contribution to formation of the Néel-like domain wall structure is can be ruled out. The resolution of the upper left region in Fig. 2a is not as ideal as other regions. This is attributed to local bending of the specimen in that small area, as manifested by the disturbed relative displacements of oxygen. In our image analysis, the unit cells in the *M*-I domain influenced strongly by the local bending on their relative displacements are circumvented.

Supplementary Note 3 The polarization-electric field hysteresis loop measurement

Supplementary Fig. 3 presents the polarization- electric field (P-E) hysteresis loop measured at room temperature for the x = 0.60 PZT crystal, which shows a typical ferroelectric hysteresis with a very high remnant polarization of 57 μC cm^{-2} and a coercive field of 44 kV cm^{-1}. These ferroelectric characteristics are consistent with the behavior expected from a tetragonal PZT composition, and attest to the high quality of the crystal. According to our estimation, the polarization value for the monoclinic and the tetragonal phase is about $P_S \approx 47$ μC cm^{-2} and 78 μC cm^{-2}, respectively. Assuming the proportion of the two structural phases is 9:11, the overall polarization for the PZT40/60 crystal is $P_S = 64$ μC cm^{-2}, which is in very good agreement with the spontaneous polarization value ($P_S \approx 62$ μC cm^{-2}) measured by the P-E loop. Therefore, considering the difference of amplitude and direction of the polarization between the two phases, the PBs with polarization discontinuity inevitably become charged owing to accumulation of either positive or negative bound charges at the boundaries.

Supplementary Note 4 Composition variation

Supplementary Fig. 4 shows that the PBs are not confined by the boundaries between regions with different composition in the phase-field simulation. This means that although the

monoclinic phase is stable and the tetragonal state metastable in x = 0.5 central region and vice versa in x = 0.6 bilateral regions, according to their bulk energies, both phases can propagate into the regions where they are metastable. Stable multiphase domain structure is dictated by minimization of energies associated with the domain and phase incompatibilities.

In our experiments, we have performed detailed composition analysis on the x = 0.60 PZT crystals. Together with the nominal composition proportion, the result averaged from dozens of detection spots using the energy dispersive X-ray spectroscopy technique is summarized in Supplementary Fig. 5. One can see that with respect to the nominal $Pb(Zr_{0.40}Ti_{0.60})O_3$ composition proportion, the proportion of Zr and Ti in the x = 0.60 crystals is increased by 0.02 and reduced by 0.05, respectively. Averagely, the standard deviation for these two elements is 17% with respect to their mean values. The composition variation detected in the x = 0.60 crystals, to a certain degree, rationalizes the composition variation implemented in the phase field simulation.

Supplementary References

1. Porta, M. & Lookman, T. Effects of tricritical points and morphotropic phase boundaries on the piezoelectric properties of ferroelectrics. *Phys. Rev. B* **83**, 174108 (2011).

2. Imry, Y. & Ma, S.-K. Random-field instability of the ordered state of continuous symmetry. *Phys. Rev. Lett.* **35**, 1399-1401 (1975).

3. Rossetti, G. A. & Navrotsky, A. Calorimetric investigation of tricritical behavior in tetragonal $Pb(Zr_xTi_{1-x})O_3$. *J. Solid State Chem.* **144**, 188 (1999).

4. Du, H. DMPFIT: Peak finding and nonlinear least-squares fitting in Digital Micrograph. *Ultramicroscopy*, under review (2016).

5. Zhao, Q. X. *et al.* First-principles study of the electronic structure and spontaneous polarization of $PbZr_{0.4}Ti_{0.6}O_3$. *Adv. Mater. Res.* **79-82**, 1249-1252 (2009).

6. Wu, Z. & Krakauer, H. First-principles calculations of piezoelectricity and polarization rotation in $Pb(Zr_{0.5}Ti_{0.5})O_3$. *Phys. Rev. B* **68**, 014112 (2003).
